# Philemon and Baucis death: a literature review

**DOI:** 10.1007/s00414-023-03126-7

**Published:** 2023-11-20

**Authors:** Lukas Sottmann, Andreas Schmeling

**Affiliations:** Institute of Legal Medicine, University Hospital Münster, Münster, Germany

**Keywords:** Philemon and Baucis death, Double death, Natural death, Sudden cardiac death, Stress

## Abstract

Double death, i.e., two bodies at a scene, is relatively rare. The double death from natural causes of two close persons is called Philemon and Baucis death in the medicolegal literature. Despite being mentioned for the first time more than 50 years ago, all detailed case reports on this case constellation are from the last 15 years. A comprehensive review of the literature on this phenomenon has not yet been published. In this article, we review the available literature on Philemon and Baucis death. Pathophysiologically, it can be assumed that this phenomenon is a sub-form of so-called psychogenic death. Therefore, we equally review the literature on sudden cardiac death due to acute psychological stress.

## Introduction

Double and multiple deaths (i.e., two or more bodies) at a scene are relatively rare and immediately arouse special attention and suspicion of non-natural death among investigators. The following constellations have to be considered: homicide, homicide-suicide (i.e., homicide with subsequent suicide of the perpetrator), double or mass suicide, extended suicide, suicide after having found a dead person (from natural causes, suicide, or homicide), accidents (e.g., traffic, electrical or industrial accident, hypothermia, fire, disaster), and intoxications (e.g., CO, food) [1].

In comparison, however, a double death from natural causes is much rarer [1–5]. In a retrospective evaluation of 5677 necropsy protocols, Thomsen et al. [5] found 6 cases of double death from natural causes in a total of 72 cases of multiple body finds. Likewise, cases in which a person commits suicide after finding a dead person from natural causes are possible and have been published [1], as well as a homicide with the natural death of the perpetrator following closely behind [2, 5]. Only after an autopsy and, if necessary, extensive further examinations can these case constellations be distinguished beyond doubt and false suspicions and frustrated investigations be prevented.

The terminus”Philemon and Baucis death” is used in medicolegal literature for the double death of two close persons, both from natural causes. This terminus stems from Greek mythology: Philemon and his wife Baucis, because of their hospitality and faithfulness to Zeus, are granted their wish to die at the same hour so that neither of them has to bury the other.

We review here the available literature on Philemon and Baucis death. Pathophysiologically, it can be assumed that this phenomenon is a sub-form of so-called psychogenic death. Therefore, we equally review the literature on sudden cardiac death due to acute psychological stress.

## Review of the literature

We performed an extensive literature research on Philemon and Baucis death and sudden cardiac death due to acute psychological stress in the relevant medical research databases (PubMed, Web of Science, Google Scholar) as of 14/07/2023. Further, we performed a backward research within the so-found relevant publications.

## Philemon and Baucis death

To date, a total of 12 cases have been reported in nine publications. The detailed results of our literature review are to be found in Table 1 of the present article.

**Table 1:** Evaluation of the literature on Philemon and Baucis death

Author/s (year of publication) [reference number]	Case number	Relationship status	Age (in years), sex (m/f), pre-existing illnesses	Spatial relationship	Postmortem interval (PMI)/condition of the corpses	Selected autopsy findings	Additional examinations (histology (H), toxicology (T), biochemistry (B))	Causes of death	Sequence of deaths
Schwarz (1970) [6]	1	Married couple	**a**: 72 m, dizziness and weakness attacks; **b**: 71 f, unspecified heart disease	Same room	PMI: 36—48 h	**a** and** b**: NS, apart from postmortem lividity fixed, intense, dark in color	**H**: NS; **T**: NS; **B**: NS	**a**: Heart failure; **b**: cardiac death	(**a**) First
Tsokos et al. (2000) [1]	2	Siblings/sisters (lived together)	**a**: 69 f, NS; **b**: 89 f, NS	Same room	NS	**a**: NS; **b**: NS	**H**: NS; **T**: negative; **B**: NS	**a**: Heart attack; **b**: heart attack	Not determinable
Ciesiolka et al. (2007) [7]	3	Married couple	**a**: 70 m, NS; **b**: 62 f, diabetic angiopathia, two below-knee amputations	Different floors of one house	PMI: max. 1 d	**a**: Pericardial tamponade (300 ml), rupture of the left ventricular posterior wall, subacute myocardial infarction, subacute coronary thrombosis of LCx; **b**: heart weight of 508 g, fibrotic myocardium, coronary sclerosis (completely blocked stent in the LAD)	**H**: Performed; **T**: a: BAC 0.44 per mill, b: 0.42 per mill, both: no other substances detected; **B**: NS	**a**: Subacute myocardial infarct with pericardial tamponade; **b**: acute uncompensated chronic congestive heart	Not determinable
	4	Married couple	**a**: 85 m, NS; **b**: 75 f, reduced mobility	Same room	PMI: min. 8 d, max. 15 d; (**a**: further advanced) signs of decomposition, (**a**: further developed) maggots	**a**: Coronary sclerosis (> 80% stenosis of LAD), myocardial scars; **b**: blood in the entire esophagus, stomach and colon, variously aged gastric mucosal erosions	**H**: Performed; **T**: a: BAC 0.20 per mill, b: BAC 0.19 per mill, both: otherwise negative; **B**: NS	**a**: Acute left ventricular heart failure; **b**: hypovolemic shock due to acute gastrointestinal bleeding from recurrent gastric mucosal erosion	(**a**) First
Delannoy et al. (2013) [11]	5	Married couple	**a**: 81 m, NS; **b**: 84 f, Alzheimer-type dementia	Same room	early state of decomposition	**a**: Multiple organ failure, fracture of the left femoral neck bone, old myocardial lesions of the left ventricle, atherosclerotic obstruction (80% in coronaries, 50% in carotids); **b**: coronary obstruction (up to 50%), myocardial ischemic sequelae	**H**: Performed; **T**: negative for toxic substances, alcohol, and carbon monoxide (CO); **B**: NS	**a**: Polyvisceral failure; **b**: probably cardiac cause	NS
	6	Married couple	**a**: 85 f, NS; **b**: 88 m, diabetes melliltus, atrial fibrillation, left ventricular failure	Same room	PMI: approx. 1 week; early state of decomposition	**a**: Ischemic heart disease with large areas of old ischemia of the left ventricle and septum, coronary obstruction and stenosis, dilated cardiomyopathy; **b**: restrictive cardiomyopathy attributed to amyloidal deposits, advanced atherosclerosis	**H**: Performed; **T**: negative for toxic substances, alcohol, and carbon monoxide (CO); **B**: NS	**a**: Heart attack; **b**: probably rhythm disorder	NS
	7	Married couple	**a**: 85 m, hypertension, ischemic heart disease; **b**: 83 f, atrial fibrillation, depression	Same room	PMI: max. 48 h	**a**: Multiple ischemic sequelae of the left ventricle, blocked coronary arteries (up to 80%), ischemic heart disease with large areas of old ischemia; **b**: dilated cardiomyopathy, coronary atherosclerosis without obstruction	**H**: Performed; **T**: negative for toxic substances, alcohol, and carbon monoxide (CO); **B**: NS	**a**: Heart attack; **b**: probably rhythm disorder	NS
Zivkovic et al. (2014) [9]	8	Siblings (lived together)	**a**: 63 m, chronic alcoholism, reduced mobility, amputated lower leg; **b**: 65 m, chronic alcoholism, reduced mobility, degenerative hip disease	Same room	PMI: max. approx. 3 weeks; partial mummification, putrefaction incl. all organs	**a**: Wischnewsky spots; **b**: Wischnewsky spots	**H**: NS; **T**: negative; **B**: NS	**a**: Not determinable (terminal hypothermia); **b**: not determinable (terminal hypothermia)	NS
Lardi et al. (2014) [8]	9	Married couple	**a**: 61 m, COPD, diabetes mellitus, depression; **b**: 63 f, alcoholism	Different rooms; **a**: bathroom; **b**: bedroom	PMI: max. 5 days; advanced decompositional changes	**a**: Mild to severe atherosclerosis incl. coronary arteries, pulmonary emphysema; **b**: mild to severe atherosclerosis incl. coronary arteries	**H**: Performed (in addition, neuropathology); **T**: a: medication within therapeutic range, b: negative, but chronic alcohol consumption in the last 3–4 months (hair); **B**: performed; pm radiology performed	**a**: Natural causes; **b**: natural causes	Not determinable
	10	Father and son (lived together)	**a**: 89 m, polymyalgia rheumatica, hypertension; **b**: 67 m, NS	Same room	Decompositional changes and beginning mummification	**a**: Partial stenosis of the RCA, cardiac hypertrophy and dilatation, heart weight of 510 g; **b**: pulmonary embolism and infarction, right atrium dilatation, deep vein thromboses of different ages, heart weight of 500 g	**H**: Performed (in addition, neuropathology); **T**: negative, but chronic alcohol consumption in the last 3–4 months (hair); **B**: performed; pm radiology performed	**a**: Ketoacidosis in association with ischemic heart disease; **b**: natural causes (ischemic heart disease, pulmonary embolism and pulmonary infarction)	Not determinable
Bondari et al. (2017) [12], Bondarev et al. (2018) [53]	11	Married couple	**a**: 60 f, alcohol abuse; **b**: 63 m, ischemic stroke, walking disability	Adjacent rooms; **a**: “cold cellar, connected to the kitchen,” **b**: kitchen	NS	**a**: Signs of hypothermia (pinkish lividities, Wischnewsky spots, hemorrhagic pancreonecrosis); **b**: recent myocardial infarction, coronary sclerosis and pronounced stenosis, old ischemic stroke	**H**: NS; **T**: a: BAC 3.5 per mill, b: no alcohol, both: further toxicology NS; **B**: NS	**a**: Hypothermia; **b**: acute myocardial infarction	(**a**) First
Tettamanti et al. (2021) [10]	12	Siblings (lived together)	**a**: 68 f, visually impaired; **b**: 60 m, vascular hypertension, schizo-affective psychosis	Same room	PMI: 5–6 days; (**a**: further) advanced putrefaction	**a**: Pathological thinness, generalized muscular atrophy, advanced putrefactive changes including all organs and cavities; **b**: doubtful myocardial area in the back wall of the left ventricle, three-vessel coronary sclerosis, myocardial hypertrophy and dilatation	**H**: Performed;** T**: negative (for drug consumption); **B**: performed (ketoacidosis excluded)	**a**: Natural causes with hypothesis of septic contribution; **b**: myocardial infarction	(**a**) First

Abbreviations: *BAC*, blood alcohol concentration; *LAD*, left anterior descending artery; *LCx*, left circumflex artery; *NS*, not specified; *RCA*, right coronary artery

### Definition

According to our literature review, Schwarz was the first to use the terminus “Philemon and Baucis death” in the medicolegal literature in 1970. He writes: “Double death from natural causes is a very rare occurrence. We see it, for example, in old married couples who were sickly and die shortly after each other. We may then speak of a Philemon and Baucis death [translated by the authors]” [6].

Subsequently, this designation was used again in 1977 in a retrospective evaluation of autopsies by Bauer [3]. However, it is not clear from the publication whether any cases listed were considered Philemon and Baucis deaths by Bauer.

In another retrospective work, a case was reported under the designation “Baucis death” by Tsokos et al. [1]. In contrast to the example by Schwarz [6] and to the constellation in the eponymous Greek myth, this case involves a brother and sister, and not a married couple. The definition is also expanded here to the extent that multiple deaths are included—in contrast to the limitation to double deaths by Schwarz [6]. Furthermore, the definition of Tsokos et al. [1] seems to make a very strict requirement regarding the temporal connection of the deaths—it presupposes “simultaneous” death. In contrast to this criterion, Tsokos et al. [1] place a sudden death from internal cause, from excitement, upon finding the corpse of a close person. The latter constellation is explicitly assigned to the Philemon and Baucis syndrome by authors of more recent works [7–12].

### Age of the deceased

Philemon and Baucis death predominantly affects old people. The mean age of all involved individuals is 73.7 ± 10.6 years. All reported cases are in individuals above 60 years of age.

### Scene of death

In the literature, bodies are mostly found in the same room (9 of 12 cases [1, 6–11]) or, less frequently, in different rooms of one apartment or house (3 cases [7, 8, 12]). A close local connection of the deaths is therefore a central common feature of the cases.

### PMI

Only one case with a PMI < 24 h has been published so far [7], and only two further cases with a PMI ≤ 48 days [6, 11], while the vast majority of reports involves corpses with advanced decompositional changes. The isolation in which the deceased often lived together has been reported as a possible explanation for the typically rather late discovery in Philemon and Baucis death [9, 10]. In many cases, this results in limited assessability and complicates the diagnosis of an internal disease as the cause of death [8, 9].

### Pre-existing diseases and causes of death

With regard to the results of the autopsy, almost all case reports found cardiac disease in both (8 cases [1, 7, 8, 11, 12]) or at least one of the deceased (3 cases [6, 8, 10]). Only in one case with far advanced putrefaction and thus severely limited assessability, no heart disease was diagnosed [9]. In exceptional cases, however, the triggered psychological stress might also lead to death via a non-cardiac previous illness or at least favor it, as reported in a case by Ciesiolka et al. (acute gastrointestinal bleeding from recurrent gastric mucosal erosion) [7].

Other repeatedly reported pre-existing conditions are diabetes (3 cases/3 individuals [7, 8, 11]), hypertension (3 cases/3 individuals [8, 10, 11]), limited mobility (4 cases/5 individuals, [7, 9, 12]), and various mental illnesses (6 cases/8 individuals [8–12]), including alcoholism (3 cases/4 individuals [8, 9, 12]).

Limited mobility and mental illness are factors that can lead to social isolation and dependence on the close one [10], the latter have been reported in a remarkable proportion of cases in the literature (*n* = 8 [7, 9–12]).

### Sequence of the deaths

In one third of the published cases, the authors established a sequence in which the deaths have occurred in consideration of the condition of the corpses, autopsy findings, and criminal investigations. In most cases, however, no sequence has been established.

### Additional examinations

In the literature, additional examinations are mostly very limited, partly due to the often encountered advanced putrefactive changes of the bodies. Toxicological examinations were reported in most [1, 7–12], but not all publications [6]. Histological examinations are reported in only a few publications [7, 8, 10, 11], while biochemical examinations have been performed in only two publications [8, 10].

## Sudden cardiac death due to acute psychological stress

The death of a close person is one of the most potent psychological stressors [13]. It is associated with disruptions of life routines, financial security, and social support [13]. The surviving person carries an increased risk of also dying in the subsequent period—a phenomenon known in epidemiology as the widowhood effect, which is most pronounced in the first months after widowhood [14]. It has been shown that even hospitalization of one spouse is associated with an increased risk of death for the partner [15].

### Increasing cardiac mortality following acute psychological stress

Brenn and Ytterstad reported higher cardiac mortality in women in the first week of widowhood compared to a control group [16]. In many epidemiological studies, increased cardiac mortality has been described after earthquakes, outbrakes of war, and important football matches and attributed to acute psychological stress as the main cause [17–19].

Case studies have linked acute intense emotions such as anger, fear, and grief to subsequent sudden death [20, 21]. In one such study 50 years ago, Engel analyzed the stories of 170 sudden deaths in the context of stressful or disrupting life events [21, 22]. Interestingly, men died more often in response to danger, whereas sudden death in women occurred more often in response to the death of a close person according to this publication [21, 22].

There is now—50 years later—increasing evidence for an association between acute emotional stress and sudden cardiac death [23]. The literature ranges widely from the abovementioned anecdotal reports and epidemiological data to experimental animal models and human studies which will be focussed on in the following section [23].

Acute emotional stress has potentially fatal adverse effects on the heart by causing disturbances of cardiac rhythm, myocardial ischemia, or left ventricular contractile dysfunction [22, 24]. It is estimated that at least 20% of sudden cardiac deaths are preceded by intense emotional stress [20, 25, 26].

Long known is the proarrhythmic effect of the sympathetic nervous system on the heart, which can trigger ventricular arrhythmias [27]. The capabilities of functional neuroimaging have recently allowed deeper insights into the neurophysiological processes by which emotional stress affects cardiac function and may ultimately lead to cardiovascular events [22, 23]. Several brain and brain stem regions process psychological stress and are linked to the heart via efferent and afferent autonomic nerves [23]. In the heart, sympathetic and parasympathetic nerves directly modulate myocardial perfusion (by epicardial coronary artery and microvascular tone) and cardiac electrophysiology [23].

### Disturbances of cardiac rhythm

Emotional stress leads to a lateralization of brain activity [22]. According to recent research, this could lead to asymmetric stimulation of the heart and areas of inhomogeneous ventricular repolarization and thus a breeding ground for ventricular arrhythmias [28]. Experimental studies showed T-wave alternans [29] and ventricular tachycardia [30], both signs of electrical instability, in response to psychological and emotional stress. Termination of potentially fatal arrhythmias has been shown to be more difficult in stressed animals [31]. This suggests that acute psychological and emotional stress may cause sudden cardiac death by inducing lethal ventricular arrhythmias [30].

### Myocardial ischemia

Psychological stress produces significant increases in heart rate and blood pressure that lead to increased myocardial oxygen demand [32]. At the same time, in contrast to the decrease during physical stress, systemic vascular resistance increases during emotional stress [33]. Indeed, mental stress can induce myocardial ischemia in individuals with coronary artery disease even if exercise or chemical nuclear stress tests were negative [34]. Remarkably, mental stress-induced ischemia is associated with higher rates of subsequent fatal and nonfatal cardiac events compared to exercise-induced ischemia [35]. Patients with underlying coronary artery disease are at special risk as impaired dilatation and even vasoconstriction under psychological stress have been described for them, opposed to coronary artery dilatation in healthy patients [36].

In addition, evidence from the literature indicates that acute psychological stress leads to an increased risk of rupture of vulnerable atherosclerotic plaques [32], causing myocardial infarction [37].

### Left ventricular contractile dysfunction

Stress cardiomyopathy (also named takotsubo cardiomyopathy, transient left ventricular apical balooning, or, interestingly, broken heart syndrome) is an acute onset, often reversible but potentially lethal, severe dysfunction of the myocardium that primarily affects the left ventricle [38, 39]. It is believed to be an underlying aetiology of sudden cardiac death subsequent to stress [40, 41]. The symptoms correspond to an acute coronary syndrome (sudden chest pain, shortness of breath). It most commonly occurs in elderly women and shortly after intense emotional or physical stress [38, 39, 42, 43]. The release of high levels of catecholamines during acute stress is currently considered the most likely cause of the acute left ventricular dysfunction [22]. This release of catecholamines could lead to microvasular coronary vasospasm [38] and myocardial stunning [39], particularly at the cardiac apex, which has a greater density of beta-adrenergic receptors [44].

### Predisposing factors

Sudden deaths primarily affect individuals with heart disease. Only in 10–20% of all sudden deaths no structural cardiac abnormalities are present [45]. Important potential causes in these cases are channelopathies—such as Brugada syndrome, long QT syndrome, or catecholaminergic polymorphic ventricular tachycardia (CPVT)—for which again stress-induced changes in the autonomic nervous system have been shown to induce lethal arrhythmias [23].

An autopsy study by Lecomte et al. showed that stress-related sudden death primarily affects individuals with severe heart disease (40 of 43 cases), especially coronary heart disease [46]. Fibrosis and/or cardiomegaly were encountered in almost all cases, both of which can lead to electrical instability [46]. Albrecht et al. [2] stated that an organically healthy person cannot die solely due to a psychologically exceptional situation. Nevertheless, young individuals without cardiovascular pathology (3 of 43 cases) were also affected, though channelopathies have not been examined [46]. Another study of 110 sudden cardiac deaths during or immediately after a stressful event (noteworthy, primarily restraint and altercation) in a predominately young cohort with a mean age of 36 ± 16 years showed a “negative autopsy and a morphologically normal heart” in 53% of the cases [37].

Furthermore, social and psychosocial determinants have been associated with sudden cardiac death including death of a significant other within the last 6 months [47], psychiatric illness [47, 48], social isolation [48, 49], and a low level of education [48, 50].

## Discussion

Our literature review yielded 12 published cases of Philemon and Baucis death. Despite being mentioned for the first time more than 50 years ago, all detailed case reports on this forensic case constellation originate from the last 15 years [7–11]. A comprehensive review of the available literature on this phenomenon has not yet been published.

With regard to the case definition and the criteria of Philemon and Baucis death, there are similarities and differences.

All authors, with the exception of Tsokos et al. [1], speak of Philemon and Baucis death only in double death cases; multiple deaths are thus not included.

For all authors, the focus of the case definition is that both deceased persons died from natural causes. Some authors explicitly refer the phenomenon only to married couples, matching both the eponymous Greek myth of Philemon and Baucis and the first reported case of this phenomenon in the medicolegal literature by Schwarz [6]. While most published cases are indeed those of married couples (*n* = 8), also 3 cases of siblings [1, 9, 10] and one case with a father-son constellation [8] have been reported and referred to as Philemon and Baucis death. Individual authors make further specifications on the connection of the deceased: cohabitation [9, 11, 12] and an emotional connection [9].

Many authors name a close temporal connection of the deaths as another central criterion [1, 3, 6, 7, 9, 11, 12, 51]. From a pathomechanistic perspective, the second individual should die close in time to either witnessing the death or finding the body of the first deceased. However, this means that potentially there could be a longer interval between the two deaths if the discovery of the corpse of the first deceased is delayed. A local connection of the deaths is also explicitly named as a criterion by some [7, 9, 11, 12].

After reviewing the literature, the following defintion can be given: a Philemon and Baucis death is the double death (i.e., both bodies are found at the same time and location) from natural causes of two persons who were connected in a special way and who died in close temporal relation.

All conceivable combinations of the natural death of one person with the non-natural death of another person are excluded, such as in cases of a suicide after the natural death of a close person. Also excluded are cases in which the finding of a deceased person from a non-natural cause (e.g., homicide, suicide, accident) is followed by the natural death of the finder, even though it can be hypothesized that the finder’s death is based on the same pathomechanism as in Philemon and Baucis death. Against this background, the assignment of a case with a non-natural death of one individual (blood alcohol concentration of 3.5 per mille, Wischnewski spots, lying in a cold cellar) to Philemon and Baucis death by Bondari et al. [12] appears inapplicable, even if other criteria may apply.

In our opinion, moreover, the term should not be used for deaths that are most likely to occur at the same time purely by chance, such as in the case of bedfellows in hospital [4]. Simultaneous deaths of children from genetic causes, as reported in simultaneous SIDS [52], or due to contagious diseases should also not be included.

Elderly people seem to be particularly at risk of dying in response to the death of a close one. This might—in accordance with the proposed pathomechanism—be explained by the higher prevalence of internal diseases and, in particular, of cardiac pre-damage with increasing age as a presumed disposition in Philemon and Baucis death. Further explanations might be the higher degree of dependence on one another and the longer developed relationship between the deceased in old age, with the consequence that the psychological stress caused by the death of the close person would thus be significantly greater in the elderly.

Today, there exists strong evidence in the literature that psychological stress may evoke severe and potentially lethal cardiac adverse events such as arrhythmia, myocardial ischemia or left ventricular contractile dysfunction. Sudden cardiac death as a reaction to the death of another person thus no longer belongs to the sphere of myths [23]. On the contrary, it is a scientifically plausible explanation for Philemon and Baucis death, especially in those with pre-existing cardiac pathology.

Besides pre-existing heart disease, psychiatric illness [47, 48], social isolation [48, 49], and a low level of education have been associated with sudden cardiac death. Remarkably, mental illness and social isolation have also been described repeatedly by different authors in Philemon and Baucis death.

For the correct diagnosis of a Philemon and Baucis death, the exclusion of non-natural causes of death is mandatory. Therefore, even if there is no suspicion of an external cause of death, a Philemon and Baucis death must not be prematurely considered to be proven. If a natural cause of death for both deceased persons cannot be established beyond doubt during the autopsy (which is the rule according to our evaluation of the literature and our own experience), additional examinations should be carried out. A comprehensive toxicological examination always seems to be mandatory from our point of view, as can be seen from the above differential diagnostic considerations. The same is usually true for histological examinations. Whenever possible with regard to the condition of the corpses, biochemical examinations of the sugar metabolism should be carried out in our view, at least if no other cause of death has been established.

## Conclusion

While double death is already a rare phenomenon in forensic medicine, only a few case reports on Philemon and Baucis death have been published to date. As a link between acute psychological stress and impaired heart function—causing disturbances of cardiac rhythm, myocardial ischemia, or left ventricular contractile dysfunction—has been established in the last decades, there exists nowadays a sound scientific base on which one can argue in favor of the existence of a natural death being caused by the loss of a close person.

## Data Availability

N/a.
